# TRENDS IN DRUG-DRUG INTERACTIONS AMONG US OLDER ADULTS, 2015-2016 VS. 2021-2023

**DOI:** 10.1093/geroni/igae098.4360

**Published:** 2024-12-31

**Authors:** Dima Qato, Shyam Krishnan Ondanat Veetil, Jocelyn Wilder

**Affiliations:** University of Southern California, Los Angeles, California, United States; University of Southern California, Los Angeles, California, United States; NORC, Chicago, Illinois, United States

## Abstract

**Background:**

Adverse drug events due to drug-drug interactions are a leading cause of preventable mortality in older adults yet current information on the use of interacting regimens is limited. In this study, we quantify changes in the frequency and types of potential major (contraindicated and generally avoid) drug-drug interactions among older US adults.

**Methods:**

Descriptive analyses of a longitudinal, nationally representative sample of community-dwelling older adults in the U.S. In-home interviews with medication logs were conducted in 2015-2016 and 2021-2023. We used Micromedex to identify potential major drug-drug interactions. Primary outcomes were the prevalence of the concurrent use of medications with a major drug-drug interactions overall and for specific medications.

**Results:**

The study comprised of 4175 participants in 2015-2016 and 2819 in 2021-2023. In 2015-2016, 12.3% of older adults were at risk for a major drug-drug interaction compared with 10.8% in 2021-2023(P =.051). While declines in the use of interacting regimens involving aspirin (3.1% to 1.7%), warfarin (1.3% to 0.4%), and hydrocodone (0.9% to 0.4%) were observed during this time period, increases in the use of citalopram (0.9% to 1.4%), tizanidine (0.2% to 0.6%) and albuterol (0.3% to 0.6%) in interacting combinations was also found (all P< 0.05). Conclusions: Although the use of interacting medications among older adults has modestly declined since 2015, 1 in 9 is still potentially at risk for a major drug-drug interaction. Improving patient awareness of commonly used drug-drug interactions has the potential to reduce preventable adverse drug events associated with their use among older adults.

